# Snapshot reflection of the seasonal resilience and diversity of fungal phylotypes in the tropical Ikogosi spring

**DOI:** 10.1007/s11356-026-37829-2

**Published:** 2026-05-14

**Authors:** Deborah Ebunoluwa Adedire, Abiodun Anthony Onilude, Olubusola Ayoola Odeniyi, Oyekanmi Nash, Khomotso Semenya, John Onolame Unuofin

**Affiliations:** 1 Department of Microbiology, University of Ibadan, Ibadan, Nigeria; 2 NABDA Southwest Center, University of Ibadan, Ibadan, Nigeria; 3 Department of Environmental Sciences, College of Agriculture and Environmental Sciences, University of South Africa, Florida Campus, Roodepoort, Gauteng South Africa

**Keywords:** Water and sediment fungal diversity, Redundancy analysis, Physicochemical factors, Microbiome analysis, High-throughput sequencing

## Abstract

Freshwater ecosystems like rivers, streams, and springs harbour diverse microbial communities, including fungal and bacterial phylotypes. These communities are an important part of the aquatic ecosystem, playing key roles in biogeochemical cycles. However, research on the seasonal differences concerning the fungal diversity of Ikogosi Warm Spring’s sediments and water has been lacking. In this pilot study, we aimed to bridge this gap by employing high-throughput DNA sequencing to examine the fungal microbiome of this spring during the wet and dry seasons. Metagenomic DNA was extracted from water and sediment samples from different locations of the spring, and the fungal ITS1 region was sequenced using Illumina HiSeq technology. Sequences were processed with the DADA2 pipeline in R, enabling comprehensive taxonomic and diversity analyses. In addition, the spring’s sediment and water physicochemical characteristics were assessed, and the impact of environmental variables on fungal communities was examined using redundancy analysis. Taxonomic analysis revealed that the spring was dominated by Ascomycota and Basidiomycota, irrespective of seasonal differences. In water samples, Ascomycota represented 62.0% (wet season) and 89.0% (dry season), while Basidiomycota accounted for 37.7% and 10.7%, respectively. Sediments exhibited a similar dominance, with Ascomycota comprising 65.1% in both seasons and Basidiomycota contributing 34.8% (wet season) and 33.5% (dry season). Alpha diversity indices indicated that fungal diversity was higher during the dry season than in the wet season, with no significant difference at *p* < 0.05. Redundancy analysis showed that some physicochemical factors, such as potassium and sulphate ions in water samples, were associated with seasonal patterns. These factors also influenced fungal communities in the spring, such as *Cladosporium, Trichosporon,* and* Meyerozyma.*

**Supplementary Information:**

The online version contains supplementary material available at 10.1007/s11356-026-37829-2.

## Introduction

Tropical ecosystems are often characterized by their high biodiversity and complex ecological interactions. They are sensitive to seasonal changes, especially those involving temperature, humidity, and precipitation (Branco 2019; Sheldon 2019; Peters et al. 2019). One of such tropical ecosystem whose fungal diversity has been grossly underexplored is the Ikogosi Warm Springs, located in Ekiti State, Nigeria. This spring is a groundwater source renowned for its confluence of warm (≥ 38 ºC) and cold streams (≤ 28 ºC). It is also known for its mineral richness, thus providing a rare and diverse environment that supports a complex microbial community. One unique phenomenon of the spring is the maintenance of the cold spring and warm spring’s temperature before they merge into a confluence. It is this phenomenon that has drawn researchers and tourists to this notable spring for decades. These features create distinct environmental niches, which may influence the composition and seasonal dynamics of fungal communities in the spring (Grossart et al. 2019; Coleine et al. 2022).

Fungal phylotypes refer to a community of fungi that share similar distinguishable genetic characteristics. They are pivotal to organic matter decomposition, nutrient cycling, plant health, and ecosystem stability (Nagy et al. 2017). In aquatic ecosystems, they have been presumed to facilitate the transfer of nutrients to microbes from other kingdoms and to stimulate water movement (Liu et al. 2021). However, their diversity and resilience to environmental changes in tropical ecosystems are not well understood.

In tropical regions, research has generally focused on forest ecosystems and agricultural soils, often overlooking habitats such as hot and cold springs. These environments are characterized by extreme and fluctuating conditions that may shape distinct fungal communities and support robust biodiversity (Mommer et al. 2018; Brinkmann et al. 2019; Adnan et al. 2022; Niskanen et al. 2023). Whilst temperate regions have been extensively studied for seasonal fungal dynamics, tropical spring ecosystems remain underexplored. This has created a gap in understanding how seasonality influences fungal diversity and community resilience in these environments. Investigating fungal resilience in such fluctuating systems, particularly in response to abiotic factors like temperature and moisture variability, is essential for understanding the adaptive mechanisms that sustain these communities.

Several studies have shown that seasonal variability can affect fungal community composition, potentially fluctuating ecosystem dynamics and impacting biodiversity (Oita et al. 2021; Abrego et al. 2024). Understanding these changes is essential for conservation efforts and for harnessing fungal resources for biotechnological applications. However, the effects of seasonal shifts on the diversity and resilience of fungal phylotypes in tropical springs, like the Ikogosi Spring, remain largely unexplored.

To address the long-standing gap, this study employed high-throughput sequencing and bioinformatic analyses to provide the first detailed insights into the fungal ecology of Ikogosi Warm Spring—a tropical warm spring ecosystem. In light of the above, the specific aims were to (1) evaluate the physicochemical properties of the spring at two different seasons (2) characterize fungal diversity across different seasons, and (3) identify specific fungal phylotypes that demonstrate resilience to seasonal environmental changes. By evaluating fungal communities across seasons, it is presumed that this research will support future efforts to elucidate patterns of diversity, composition, and functional adaptation in tropical spring fungi. Microbiome studies have proven to be extremely useful in unravelling the microbial ecology and diversity of different natural environments (Shu et al. 2022; Mallik et al. 2024). It can also be used as an evaluative tool to address environmental changes, since change is heavily dependent on microbial responses in that particular ecosystem (Cullen et al. 2020). The novelty lies in the focus on a unique tropical spring ecosystem, where both hot and cold springs converge, creating a dynamic environment that may support unique fungal phylotypes with high resilience to environmental stressors. This study will contribute to the broader understanding of fungal ecology and adaptability in extreme environments of tropical regions. It will also provide foundational knowledge about the response of fungal communities to seasonal variations, which is critical for ecological conservation and potential biotechnological exploitation of tropical fungal resources in resource-poor tropical regions.

## Materials and methods

### Study area and sampling sites

**Fig. 1:**
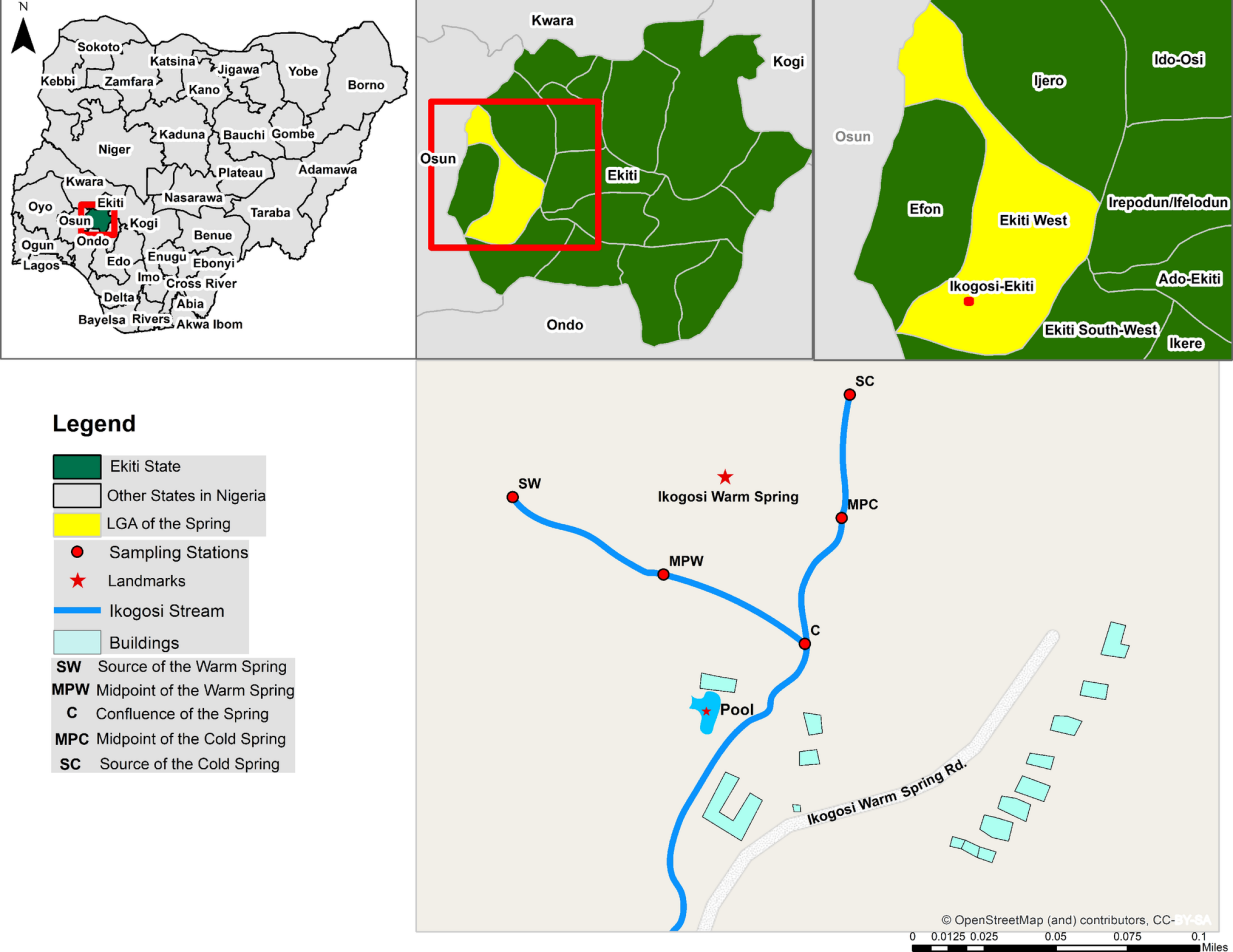
Map of the study area, Ikogosi Spring, showing its location and its sampling points

This research was conducted in Ikogosi warm springs, in Ikogosi-Ekiti Local Government Area of Ekiti State, located in the South-western region of Nigeria. The spring is used for recreational, culinary, and drinking purposes by tourists and inhabitants within its locality (Oladipo et al. 2005). The location of the warm and cold spring was recorded as (7^o^35′28.227’’N, 4^o^59′2.925’’ E). Five sampling sites were selected for the purpose of this study, designated SW (source of the warm spring), MPW (Midpoint of the warm spring), C (Confluence of the spring), MPC (Midpoint of the cold spring), and SC (source of the cold spring) and are as depicted in Fig. 1.

### Sample collection

Five sampling locations of Ikogosi warm springs were chosen following the different degrees of anthropogenic impact: SW- source of the warm spring, MPW—Midpoint of the warm spring, C—Confluence of the spring, MPC—Midpoint of the cold spring, and SC—source of the cold spring. Sediment and water samples were collected in duplicates for metagenomic and physicochemical examination. These samples were collected on two separate occasions: June (wet season) and December (dry season) in 2018. Each sample was a pool of different samples taken from a particular sampling point at a particular time, resulting in 20 composite samples during this research. The points SW and SC were located in pristine sites, away from anthropogenic activities, while points MPW and MPC were intermediates between these two extremes. Lastly, point C was located where the cold and warm springs merged and flowed downstream as a single stream. One litre of water was collected in duplicate at each sampling point using, clean sterile Schott bottles. Sediment samples were collected manually with the aid of a clean plastic container from the river bed surface. The supernatant was decanted, and 50 g of sediment were transferred into sterile vials for mycobiome analysis, while 350–450 g of sediment was placed in separate sterile ziploc bags for physicochemical analysis.

All samples were immediately stored on ice and transported to the laboratory. Physicochemical properties such as temperature and pH of the water body of each sampling site were measured and recorded in situ.

### Physicochemical analysis of water and sediment samples

Physicochemical parameters evaluated for water samples include dissolved oxygen, nitrates, sulphates, electrical conductivity, calcium, magnesium, manganese, potassium, sodium, copper, zinc, ferrous ions, total dissolved solids, temperature, and pH. Considered parameters for sediment samples were percentage nitrogen, available phosphorus, organic carbon, nitrates, sulphates, calcium, magnesium, potassium, sodium, manganese, copper, zinc and ferrous ions. Physicochemical parameters of all samples were investigated using the standard protocol stated in APHA (2005). Samples were subjected to atomic absorption spectroscopy for quantitative determination of sodium, iron, zinc, copper, manganese, magnesium, potassium and calcium at ultra-low level. While the hydrogen ion concentration of water samples was measured by using a properly calibrated pH meter (HI98128 pHep® 5, Hanna Instruments, USA). The temperature of water samples was also measured using a mercury thermometer (H-B Instrument Durac ASTM, USA), and recorded in °C. Electrical conductivity of water samples was measured using a conductivity meter (Fisher Scientific, USA). To determine the total dissolved solids in water samples, water samples were agitated and transferred into a filtering flask containing well-dried pre-weighed glass fibres (dried at 103 °C). Thereafter, these samples were filtered through a pump, and the filter in the filtering flask was recovered, allowed to dry at 103 °C, cooled and reweighed. The difference in the weight of the glass fibre was calculated as the total dissolved solids. All physicochemical examinations (except for pH and temperature) were performed at the Soil Microbiology Laboratory, International Institute of Tropical Agriculture, Ibadan, Nigeria. Recorded values (in duplicates) were subjected to analysis of variance. Mean physicochemical values were separated using the Duncan’s Multiple Range Test (DMRT) at 5% level of significance, using the SPSS package (version 25.0).

### Total genomic DNA extraction

#### Total genomic DNA extraction from water samples

With the aid of a vacuum pump, all water samples were passed through 47 mm membranes with a porosity of 0.2 micron. These membranes were thereafter subjected to total DNA extraction using the Quick DNA Fecal/Soil Microbe Miniprep kit (Zymo Research, USA), following the manufacturer’s instructions.

#### Total genomic DNA extraction from sediment samples

About 0.25 g of each sediment sample was dissolved in 750 µl of lysis buffer and passed through 47 mm membrane filters (with a pore size of 0.2 micron), using a vacuum pump. All membrane filters containing trapped microbial cells were subsequently subjected to total genomic DNA extraction with the Quick DNA Fecal/Soil Microbe Miniprep kit (Zymo Research, USA).

### DNA Purification, PCR amplification, library construction and sequencing

The paired index Hi-Seq protocols were employed to sequence the ITS 1 region of the fungal ribosomal genes. DNA samples were quantified using a NanoDrop Spectrophotometer 2000 (Thermo Scientific, USA) to evaluate the quality and purity of all extracted DNA. All DNA samples were also analysed on an ethidium bromide-stained 1% agarose gel. They were visualised in the gel documentation system (Biorad Gel Documentation System, USA) under UV light. 30 ng of high-quality DNA template was used for Polymerase Chain Reaction (PCR). PCR amplification was performed using the following ITS primer sets: ITS1 (5’-CTTGGTCATTTAGAGGAAGTAA-3’) (Garden and Bruns 1993) and ITS2 (5’-GCTGCGTTCTTCATCGATGC-3’) (White et al. 1993). It involved initial denaturation at 95 °C for 2 min, followed by 35 cycles of denaturation 95 °C for 30 s, annealing at 52 °C for 45 s, extension at 72 °C for 20 s sand final extension at 72 °C for 7 min.

PCR products were thereafter purified using Agencourt AMPure XP beads (Beckman Coulter, USA), and further tagged for library construction using an illumina adapter (i7) -AGATCGGAAGAGCACACGTCTGAACTCCAGTCAC. The library size and the concentration of the library were determined by Agilent 2100 Bioanalyzer (Agilent Technologies, USA). Paired-end sequencing was performed on one flow cell for 250 cycles on a HiSeq 2500 platform (Illumina Inc. USA) at BGI Genomics Co. Ltd. (Tai Po, Hong Kong).

#### Sequence analysis

Generated sequences were quality-filtered using the DADA2 microbiome pipeline (version 1.18) (Callahan et al. 2016) implemented in R (version 4.1.1) (R Core Team 2020). The following parameters for filtering were set as follows: maxN = 0, maxEE = 2, truncQ = 2, minLen = 50 and rm.phix = “true”. The fungal database—UNITE (version 8.3, accessed 20—08—2021) was used to align, classify and assign taxonomies to all sequences (Nilsson et al. 2019). Afterwards, ASVs (Amplicon Sequence Variants) assigned as mitochondria and chloroplasts were removed from the dataset. All downstream and statistical analyses were implemented in R (version 4.1.1) using the following packages: microbiomeutilities (version 1.00.16) (Lahti and Shetty 2017) for taxonomic compositional bar plots and alpha diversity computations. Phyloseq for beta diversity analyses using Bray–Curtis dissimilarity distance index (Baselga 2013; McMurdie and Holmes 2013), microbiome (version 1.14.0) (Lahti and Shetty 2017). Significant differences in computed alpha diversity metrics (Shannon, observed species, and Simpson’s) were tested using Kruskal–Wallis’s nonparametric test (McKnight and Najab 2010) using base packages available in R.

Redundancy analysis (RDA) was conducted in R using the vegan package (v2.7—1) to assess the influence of environmental variables on fungal community composition in sediment and water samples from the spring. Before analysis, data were Hellinger-transformed. Ordinations were visualised using ggplot2 (v3.5.2). For sediment samples, variables significantly associated with variation in fungal community composition were identified using one-way permutation ANOVA (999 permutations, p < 0.05). Available phosphorus, calcium (Ca), sulphate (SO₄), and organic carbon met this criterion and were used as constraining variables in the sediment RDA. For water samples, RDA was similarly restricted to environmental parameters significantly associated with community variation (p < 0.05; 999 permutations). These included temperature, total dissolved solids, potassium (K), sulphate (SO₄), and pH.

##### Metagenomics data availability

Fungal metagenome sequences generated in this study have been archived in NCBI under the SRA study ID PRJNA779450 with accession numbers: SAMN23038675—SAMN23038694.

## Results

### Physicochemical properties of sediment and water samples from different sampling points during different seasons

Duplicate water and sediment samples obtained from different points of the warm and cold springs (SW, MPW, C, MPC and SC) are shown in the supplementary section (Tables S1a and S1b).

### Summary of fungal taxonomic classification

Fungal taxonomic richness varied across sample groupings and increased gradually from higher to lower taxonomic ranks (Table S2). Overall, water samples displayed greater diversity than sediment at most taxonomic levels, mostly at the genus and species levels. Likewise, samples collected in December showed higher richness compared to those from June.

When both factors were combined, December water samples consistently showed the highest taxonomic richness, whereas June water samples showed comparatively lower values.

Across sample locations, richness increased from the warm spring source toward the cold spring locations, with the highest diversity observed at the midpoint and source of the cold spring.

The breakdown of reads before and after quality preprocessing is discussed in the supplementary section (Table S3).

### Fungal taxonomic composition of Ikogosi warm spring

**Fig. 2:**
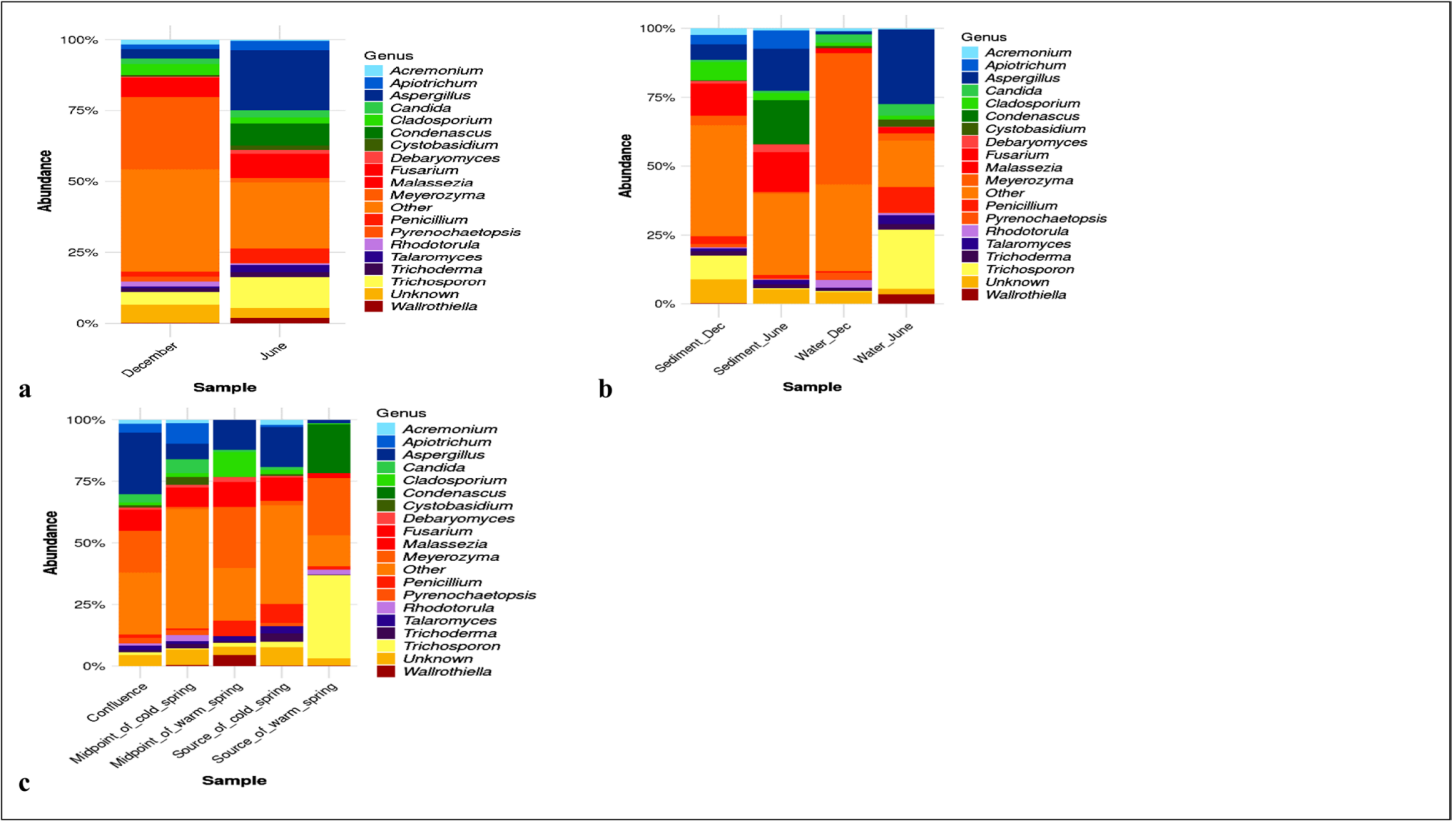
Taxonomic compositional bar plots showing the most abundant fungal genera detected from (**a**) December and June (from both sediment and water samples) from all sampling points of Ikogosi warm springs (**b**) from sediment and water samples collected in December and June (**c**) different sampling points of Ikogosi warm springs

Samples (water and sediment) collected in June (Fig. 2a), showed that there was an elevated presence of *Aspergillus* (20.51%), *Trichosporon* (15.64%), *Condenascus* (8.73%), *Malassezia* (6.88%) and *Penicillium* (5.56%) than what was observed during the dry season – December (*Aspergillus* – 3.53%, *Trichosporon*- 1.76%, *Condenascus* – 0.03%, *Malassezia*- 5.18% and *Penicillium*- 1.67%). Conversely, *Cladosporium* (4.16%), *Malassezia* (5.18%) and *Meyerozyma* (34.87%) were the most abundant genera in December samples, whereas occurrence of *Cladosporium* (2.02%) and *Meyerozyma* (1.64%) was significantly reduced in June. Unassigned ASVs were classified as unknown. The relative abundance of fungal phyla concerning seasonality is highlighted in the supplementary section (S2: Figure S1). Likewise, the relative abundance of fungal phyla and genera in water and sediment samples is shown in the supplementary section (S2: Figures S2A and S2B).

Comparing genus-level fungal diversity across seasons, SD (December sediment) was dominated by *Malassezia* (11.84%), *Cladosporium* (8.95%), and *Aspergillus* (7.36%), while SJ (June sediment) was characterised by *Condenascus* (19.42%), *Aspergillus* (14.67%), and *Malassezia* (13.53%). WD showed strong dominance of *Meyerozyma* (56.41%), followed by *Rhodotorula* (3.2%) and *Candida* (3.19%). In WJ (June water), *Trichosporon* (28.03%) was most abundant, followed by *Aspergillus* (25.28%) and *Penicillium* (9.19%) (Fig. 2b). Phylum-level composition across samples and seasons is provided in the Supplementary Material (Figure S3).

At different sampling points within the spring (Fig. 2c), *Trichosporon* (39.27%), *Meyerozyma* (25.38%), and *Condenascus* (22.79%) dominated the source of the warm spring (SW). At the source of the cold spring (SC), *Aspergillus* (18.33%), *Penicillium* (10.43%), and *Malassezia* (7.56%) were most abundant. The midpoint of the warm spring (MPW) was dominated by *Meyerozyma* (27.77%), *Aspergillus* (13.09%), *Malassezia* (8.9%), *Cladosporium* (8.6%), and *Penicillium* (6.65%), whereas the midpoint of the cold spring (MPC) was characterised by *Apiotrichum* (9.48%), *Malassezia* (6.87%), *Aspergillus* (6.82%), and *Candida* (5.96%). At the confluence point (C), *Aspergillus* (27.41%), *Meyerozyma* (19.2%), and *Malassezia* (6.95%) were most abundant. Phylum level composition across sampling points is presented in the Supplementary Material (Figure S4).

#### Fungal alpha diversity

**Fig. 3:**
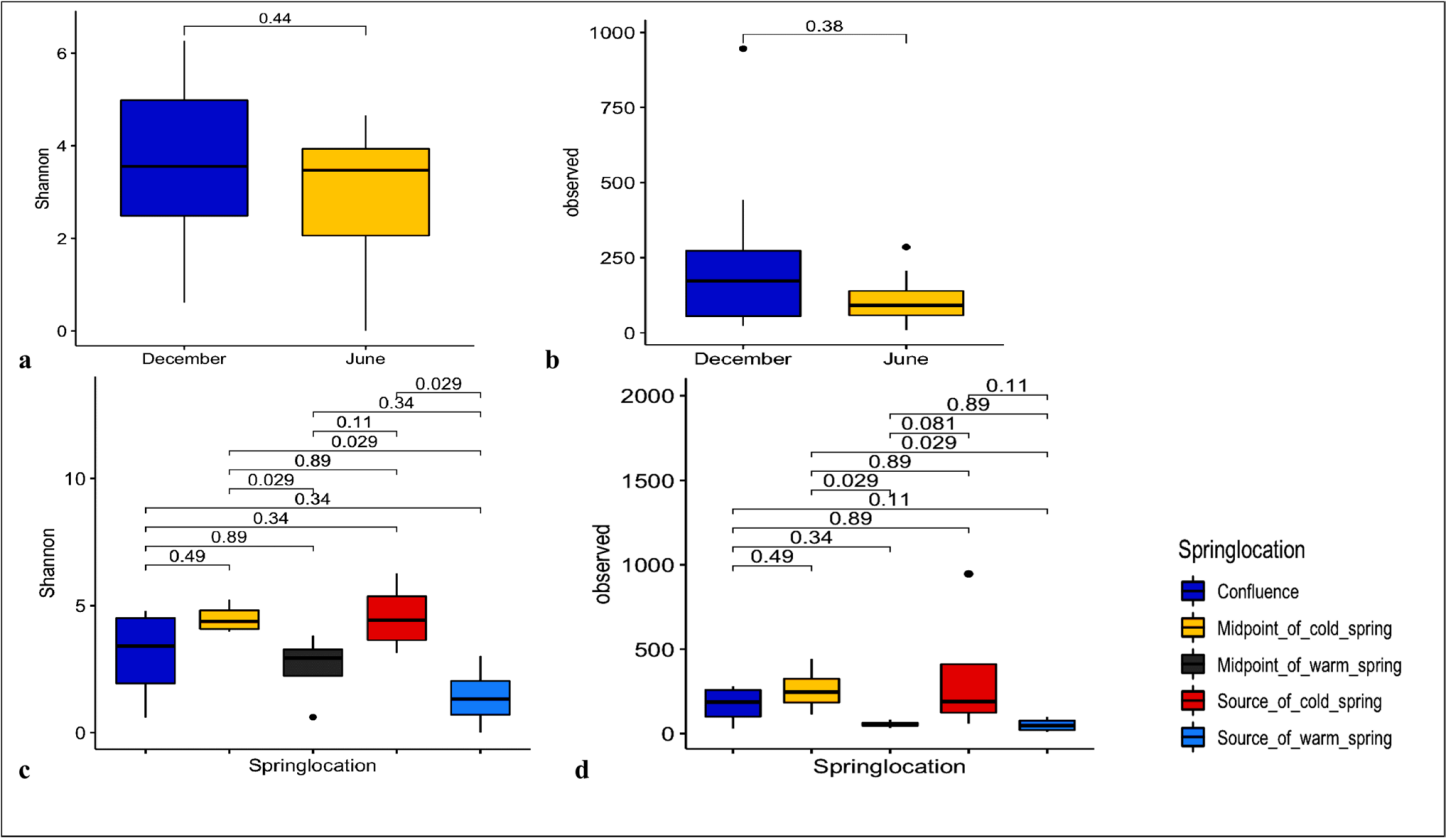
Fungal alpha diversity boxplots of (sediment and water) samples collected at different months (dry- December and wet—June) from Ikogosi warm springs using (**a**) Shannon index and (**b**) Observed species index (*P* < 0.05). Shannon and Observed species estimates showing high differences in fungal diversity between seasons: the dry (December) and wet (June) samples, with statistically insignificant p values of 0.44 and 0.38, respectively at *P* < 0.05. Fungal alpha diversity boxplots of samples collected from different sampling locations of Ikogosi warm springs combining both seasons, using (**c**) Shannon and (**d**) Observed species indices (*P* < 0.05)

The Shannon and observed species indexes were used to identify community richness (observed species) and species diversity (Shannon) between subgroups within each category (Figs. 3a–d). The boxplot showed the interquartile ranges, median, and outliers for each distribution. Statistical groupings were labelled by the numbers above the boxplots, and significance testing was done using the Kruskal–Wallis test, *P* < *0.05*. Samples collected at different points of the spring during both seasons (Fig. 3c and d) showed significant differences in fungal richness (*p* < 0.05). Both Shannon and observed species indices indicated higher diversity and richness at the midpoint of the cold spring (MPC) compared with the midpoint of the warm spring (MPW) (*p* = 0.029 for both metrics). Similarly, MPC showed significantly higher diversity than the source of the warm spring (SW) (*p* = 0.029 for both indices). The source of the cold spring (SC) also exhibited higher fungal diversity than SW. Overall, both indices indicated that samples from the midpoint and source of the warm spring had the lowest fungal diversity.

##### Redundancy analysis

**Fig. 4:**
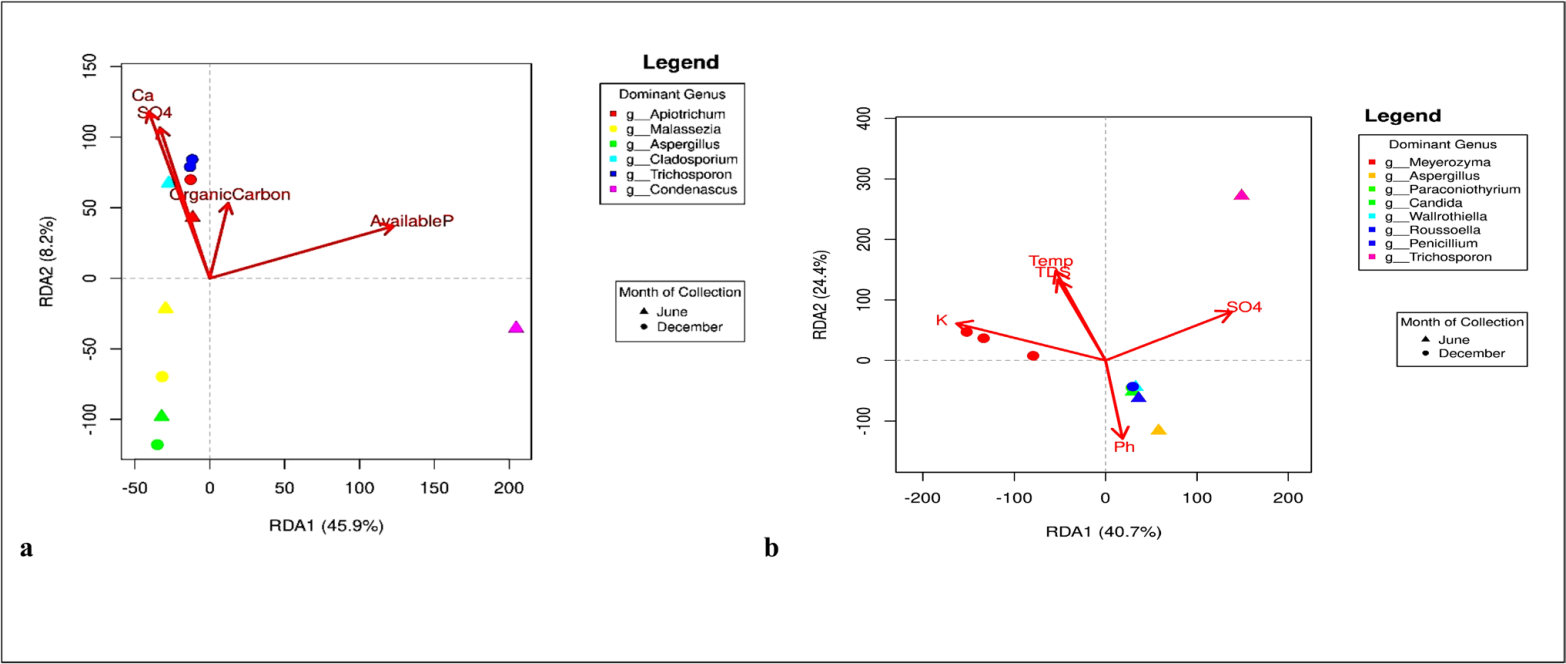
**a** Redundancy Analysis (RDA) of significant physicochemical factors on the dominant fungal genera in sediment samples collected in June and December, being the wet and dry season, respectively. The percentages of the X-axis (RDA1) and the Y-axis (RDA2) explained 45.9% and 8.2% of the observed variance, respectively. Points represent samples, coloured by dominant fungal genus and shaped by month of collection. Red arrows represent significant physicochemical factors influencing fungal community structure; **b** Redundancy Analysis (RDA) of significant environmental factors (Potassium (K), temperature, SO_4_ (sulphates), total dissolved solids (TDS), and pH) on the dominant fungal genera in water samples collected in June and December, being wet and dry season, respectively. The percentages of the X-axis (RDA1) and the Y-axis (RDA2) explained 40.7% and 24.4% of the observed variance, respectively. Points represent samples, coloured by dominant fungal genus and shaped by month of collection. Red arrows represent significant environmental variables influencing fungal community structure

Redundancy analysis (RDA) was performed to identify the potential relationships between the most significant physicochemical properties of the sediment and the dominant fungi present in sediment samples (Fig. 4a). The relationship between fungal communities in sediment samples and their corresponding environmental parameters is documented in the supplementary material (S3: Figure S5). Redundancy analysis from sediment samples (S3: Figure S5) indicated that available phosphorus was negatively correlated with most samples and showed only a weak association with dry season samples. Calcium ions were positively correlated with both wet and dry season sediments from the same spring region. However, sulphate ions were positively associated with only two sediment samples, depicting no seasonal pattern. Consistent with this, Fig. 4a showed that available phosphorus was negatively correlated with all dominant genera across samples. Calcium and sulphate ions were strongly associated with sediment samples from both seasons, dominated by *Trichosporon*, *Apiotrichum*, and *Cladosporium*. Overall, the RDA (Fig. 4a) indicated no clear seasonal structuring of sediment-associated communities. Redundancy analysis of water samples showed seasonal structuring, with distinct associations between environmental variables and community composition in the wet and dry seasons. Sulphate had the strongest influence on wet-season samples (Figure S6), where it was positively correlated with *Trichosporon* (Fig. 4b). Potassium, in contrast, was associated with dry season samples (Figure S6), with December samples clustering closely with potassium and being dominated by *Meyerozyma* (Fig. 4b).

## Discussion

In this pilot study, the impact of seasonal differences on the fungal community in Ikogosi Warm Springs was investigated. It also identified the physicochemical drivers responsible for changes in the community, and explored fungal diversity across different locations within the springs.

This was done by performing deep sequencing of the sediment and water samples from the spring. To the best of our knowledge, this is the first metagenomic study of Ikogosi warm springs, which reveals a deeper fungal structure of this popular spring in Nigeria.

ITS 1 region of the ribosomal DNA of the fungal genome was chosen as the region of choice, because of its ability to resolve closely related fungal species better than ITS2 (Lücking et al. 2021; Mbareche et al. 2020). To preserve fungal bioresources in the natural environment, it is essential to identify and characterize fungal communities appropriately using cutting-edge technologies (Hossain et al. 2025). In this study, some environmental parameters were significantly influenced by seasonality (dry and wet seasons) and sample types—sediment and water (Table S1a and b). For instance, the sediment had higher concentrations of most cations than water, but they were more bioavailable during the dry season (December), except for Na^+^ and Ca^2+^, which were more prevalent in water samples and mostly reduced in concentration during the dry season (Table S1a). Correspondingly, nitrate (NO_3_^−^) and sulphate (SO_4_^2−^) concentrations were higher in sediment than in water samples. However, whilst NO_3_^−^ and SO_4_^2−^ concentrations reduced during the dry season in water (Table S1b), concentrations of SO_4_^2−^ were elevated and NO_3_^−^ were reduced in sediment. Total dissolved solids and electronic conductivity were both high in the dry season for most sampling points along the confluence springs. Organic carbon in sediment was higher in SC and MPC during the dry season, compared to the wet season. However, it was higher in SW and C during the wet season. Nitrogen was generally higher during the dry season, across most spring locations, while available phosphorus was significantly higher in SC and SW during the wet and dry seasons, respectively.

In the cycling of materials such as cations and anions, sediments serve as a natural form of filter in waterbodies. Essential macro nutrients such as nitrates and phosphates are continuously exchanged between sediments and their overlying waters (Su et al. 2024). Analyzing the sediments of waterbodies, alongside water analysis, is increasingly essential for understanding the overall ecosystem of waterbodies (Vettorazzo et al. 2024). It can be speculated that the active interaction between the spring’s mycobiota and its environmental parameters might create a shift in the fungal diversity and the biogeochemical conditions of the spring. For instance, several fungal communities have been observed to have the capacity to influence and be shaped by physicochemical properties, such as pH, precipitation, temperature, cation exchange capacity, available phosphorus, nitrogen and organic carbon (Garcia et al. 2020; Chen et al. 2023; Li et al. 2023; Zhao et al. 2023).

The effect of seasons on the fungal structure and diversity was demonstrated by plotting taxonomic compositional bar plots (Fig. 2a). A clear differentiation was observed between fungal communities in dry and wet samples. Even though both seasons showed a high relative abundance of Ascomycota and Basidiomycota (Figure S1), the difference became obvious among the top 19 fungal genera of both seasons. For instance, *Wallrothiella* and *Condenascus* were only present in wet samples (June).

*Wallrothiella* is a genus of fungi that belongs to the phylum Ascomycota. Species of *Wallrothiella* include *Wallrothiella congregata* and *Wallrothiella subiculosa* (Huhndorfet al. 2009; Daranagama et al. 2017), none of which is naturally found in waterbodies (Daranagama et al. 2017). Further grouping revealed that *Wallrothiella* was identified from water samples collected only during the wet season (Fig. 2b). Further evaluation indicated that this genus was among the top 19 genera only at the midpoint of the warm portion of the spring (Fig. 2c). To date, there are no reports of this fungus as a sediment-dwelling organism in aquatic environments. Its occurrence in the spring water may therefore be attributed to spores or debris from nearby vegetation during the wet season.

*Condenascus*, another fungal genus detected only in samples collected during June (wet season) (Fig. 2a), was found among the top 19 genera exclusively in sediment samples (S2: Figure S2B). Further grouping of the samples showed that this fungus occurred only in sediments collected during the wet season (Fig. 2b). In addition, it was solely detected at the source of the warm section of the spring (Fig. 2c). Novak Babič et al. (2022) reported *Condenascus tortuosus* in the sands of an urban beach in Slovenia, with abundances increasing during annual monitoring, particularly from June to September. To date, *Condenascus* has not been reported in sediment or water samples from aquatic systems, even though it is recognized as a soil-dwelling fungus (Teng et al. 2024). The spring is used as a tourist site rather than for agricultural purposes, making its detection, especially at locations restricted from tourist access, particularly noteworthy. Its presence in the sediments of the warm spring is therefore intriguing and may warrant further omics study. Notably, only one species of *Condenascus* has been recognized—*Condenascus tortuosus, which was* reported in an agriculture-related study (Jalloh et al. 2024).

During the dry season, *Meyerozyma* showed higher relative abundance compared to wet season (June) samples (Fig. 2a). Taxonomic bar plots (S2: Figure S2B) indicate that this increase is driven by water samples, a pattern supported by Fig. 2b, which showed elevated abundance in water collected during the dry season (December). Figure 2c further revealed that *Meyerozyma* was more abundant in warmer regions of the spring and at the confluence where warm and cold waters mix. *Meyerozyma* is a non-pigmented ascomycetous yeast widely distributed across diverse environments (Ghasemi et al. 2022). It is an opportunistic organism capable of tolerating various stress conditions (Corte et al. 2015) and has been reported from thermal habitats, including geothermal plants and hydrothermal systems (Ramesh et al. 2021; Bregnard et al. 2024). It has also been isolated from tropical freshwater systems such as lakes and rivers (Medeiros et al. 2012; Brandão et al. 2017; Monapathi et al. 2017).

Taxonomic composition analysis of the top 19 fungal genera across sediment and water samples in different seasons (Fig. 2b) revealed clear shifts in community structure. For example, *Cladosporium* decreased in relative abundance in sediment during the wet season compared to the dry season. Likewise, *Trichosporon* showed a marked seasonal shift, with high abundance in wet-season water samples but minimal presence in the dry season. In contrast, *Aspergillus*, *Apiotrichum*, and *Malassezia* maintained relatively stable abundances across both sample types and seasons, indicating a certain degree of stability and dominance.

The occurrence of *Malassezia* and *Aspergillus* in spring sediments is expected, as microbial ecology studies have confirmed their presence in sediments, soil, and aquatic habitats. (Amend et al. 2019; da Silva et al. 2022; Lin et al. 2023). Despite the low nutrient availability in Ikogosi Springs, these taxa likely persist due to their tolerance of unfavorable conditions. The detection of *Cladosporium* and *Aspergillus* in both water and sediment is in tandem with findings from Doi et al. (2018), where these genera were similarly abundant in the water and sediment of Araçá Bay, Brazil. Similarly, with reports from Calabar, Nigeria, where *Aspergillus* was identified from a freshwater habitat (Okpako et al. 2009).

*Malassezia* (a member of the phylum Basidiomycota) is known to be ubiquitous across diverse environments, including marine and terrestrial systems, hydrothermal vents, dust, and mammalian skin (Steinbach et al. 2023; Rahimlou et al. 2025). Its presence in the tropical springs of Ikogosi may therefore reflect anthropogenic input from tourists and inhabitants living within the spring’s locality.

Alpha diversity analysis was also used to understand the underlying fungal community inhabiting the spring. Across seasons (December and June; dry and wet, respectively) (Fig. 3a), none of the alpha diversity metrics showed significant differences, although mean diversity was higher in the dry season. Likewise, comparisons between sediment and water across seasons revealed no statistically significant variation (S4: Figure S5). Overall, Shannon and observed species metrics indicated limited differences in alpha diversity, likely driven by high taxonomic overlap and possibly constrained by low sample size.

Across sampling locations (Fig. 3b), both alpha diversity indices indicated that richness, evenness, and taxonomic diversity were significantly higher at the midpoint of the warm spring (MPW) than at the midpoint of the cold spring (MPC). This pattern likely reflects differences in physicochemical conditions between these sites. MPW exhibited slightly acidic pH (5.80–6.36) and higher temperatures (up to 38.0 °C), whereas MPC showed neutral pH (7.17–7.40) and lower temperatures (29 °C). These conditions are consistent with reports that pH and temperature strongly influence microbial community structure in aquatic systems (Adedire et al. 2021).

In contrast, alpha diversity was significantly lower at MPW compared to the source of the cold spring (SC). This difference may be linked to sediment characteristics, with MPW dominated by loosely weathered sediments with low water-holding capacity, while SC consists of loamy, moisture-retentive soils that may promote microbial attachment and persistence. Temperature may also contribute, as MPW remained consistently warmer (37.3 °C, across seasons) than SC (26.67–28.33 °C, across seasons), potentially supporting higher microbial diversity at SC.

Redundancy analysis of sediment samples (Fig. 4a) showed an association between calcium and dominant fungal genera across both seasons. This suggests a role for calcium in microbial growth in the spring, independent of seasonal variation. Calcium is a key element in freshwater systems, influencing microbial proliferation and community dynamics (Weyhenmeyer et al. 2019). It is also involved in intracellular signaling and enzymatic processes (Hessen et al. 2017). The lack of seasonal separation indicates that sediment community composition was not strongly differentiated by season. Instead, it showed consistent associations with variables such as calcium and sulphate.

However, there seemed to be a seasonal influence on fungal communities from water samples, with well-defined environmental drivers, such as sulphate and potassium. Shaping the community in the wet versus dry season. Sulphate had the strongest influence on wet season water samples (S4:Figure S7). In freshwater systems, sulphates are derived from processes such as volcanic activity, sulphide oxidation, organic decomposition, and combustion of organic matter (Zak et al. 2021). During the wet season, *Trichosporon* was positively correlated with sulphates in the spring waters (Fig. 4b). The presence of sulphate may reflect organic decomposition within the spring’s ecosystem, particularly from plant debris such as leaves and shrubs. It may also result from inputs of organic matter from human activities. This is supported by reports that *Trichosporon* species can tolerate or transform organosulfur compounds and participate in sulphur metabolism within mixed communities (Yang et al. 2011; Singh et al. 2019).

Figure S7 showed that potassium influenced dry season water samples, a pattern also evident in Fig. 4b. In the dry season, the association between potassium and *Meyerozyma* suggests a potential physiological link. *Meyerozyma* is a yeast genus within Saccharomycetales (Ascomycota), comprising species such as *M. guilliermondii*, *M. carpophila*, and *M. caribbica* (Ghasemi et al. 2022). *M. guilliermondii* is a saprophyte found on human mucosa and skin. It has also been reported from freshwater, marine, and soil environments (Serra et al. 2019; Mo et al. 2024). The association between potassium and *Meyerozyma* may reflect the role of potassium in yeast physiology, including enzyme activation, cell volume regulation, and maintenance of cellular electroneutrality (Illarionov et al. 2020; Masaryk and Sychrová 2022).

The seasonal-habitat dynamics in the Ikogosi spring suggest that seasonal inputs strongly influence fungal adaptation. In the wet season, increased allochthonous material likely supports filamentous fungi capable of degrading complex lignocellulosic and humic substrates, a pattern consistent with freshwater systems receiving terrestrial runoff (Grossart et al. 2019). By contrast, the dry season appears to favor more specialized taxa, including thermophilic and oligotrophic fungi adapted to warmer, nutrient-limited conditions. These shifts demonstrate the ability of fungal communities to persist under changing resource availability and temperature regimes (Amend et al. 2019). Habitat variability within the spring further refines this distribution. Warmer submerged zones tend to support thermotolerant fungi, often forming biofilms and interacting with other microorganisms, whereas cooler transitional areas, such as the confluence with the cold spring, harbor mesophilic and plant-associated taxa, reflecting the mixing of aquatic and terrestrial influences (Hyde et al. 2016). Collectively, these seasonal and spatial patterns illustrate the ecological flexibility of fungal phylotypes in Ikogosi Spring. Their resilience is closely tied to metabolic adaptability, including the production of lignin-modifying enzymes and other secondary metabolites that might play key roles in organic matter transformation as well as cycling of inorganic materials. Overall, the fungal communities in Ikogosi Spring reflect a balance between environmental pressure and adaptive response, underscoring their importance as both ecological drivers and reservoirs of functional diversity.

## Conclusion

The fungal microbiome in Ikogosi Warm Spring waters was influenced by seasonal variation, with sulphate and potassium showing significant correlations with water samples across seasons. These findings suggest fungal communities exhibit adaptability to changing environmental conditions. This study provides baseline information on fungal phylotypes in both sediment and water, and highlights taxa with potential for industrial metabolite applications. Overlapping mycobiomes between sediment and water were also observed, supported by diversity analyses. Overall, this pilot study establishes a foundation for future research on seasonal effects on spring mycobiomes and the exploration of fungi with ecological and biotechnological relevance. To our knowledge, this is the first report on fungal diversity in Ikogosi Warm Spring, Nigeria.

## Limitations of the study

We sampled for two seasons, during a one-year period, therefore, the analyses may not be rigorous enough to predict accurate seasonal dynamics of fungal microbiomes occurring in the spring over a long period of time. A longer period of collection, in terms of extended years, can be implemented to further give wider and clearer information, in checking the stability of these taxa in the spring. Limited by funds, sample sizes were likewise limited in number. Therefore, it may not be sufficient to determine if some physicochemical factors had stronger effects than what was reported. As stated earlier, this pilot study has provided baseline and exploratory reports regarding fungal communities inhabiting the spring; however, more scientific experiment is recommended.

## Supplementary Information

Below is the link to the electronic supplementary material.

ESM 1 (DOCX 622 KB)

## Data Availability

Fungal metagenome sequences generated in this study have been archived in NCBI under the SRA study ID PRJNA779450 with accession numbers: SAMN23038675—SAMN23038694. Other sources are available as supplementary material.
