# Supplementary material for: Snapshot reflection of the seasonal resilience and diversity of fungal phylotypes in the tropical Ikogosi spring
Source: Environ Sci Pollut Res Int. 2026 May 14;33(17):8264–75. doi: 10.1007/s11356-026-37829-2 (PMC13226328; doi:10.1007/s11356-026-37829-2)
Supplement: Supplementary file 1 — (DOCX 622 KB) [file 11356_2026_37829_MOESM1_ESM.docx]

**Snapshot reflection of the seasonal resilience and diversity of fungal phylotypes in the tropical Ikogosi Spring**

Deborah E. Adedire^1,2*^, Abiodun A. Onilude^1^, Olubusola A. Odeniyi^1^, Oyekanmi Nash^1,2^, Khomotso Semenya^3^, John O. Unuofin^3*^

**Table S1a. Mean physicochemical values from sediment samples obtained at respective sampling points from Ikogosi springs in June and December, 2018**

| **WET SEASON (JUNE) DRY SEASON (DECEMBER)** | | | | | | | | | | |
| --- | --- | --- | --- | --- | --- | --- | --- | --- | --- | --- |
|  | **SAMPLE SITES** | | | | | **SAMPLE SITES** | | | | |
| Parameter | SW | MPW | C | MPC | SC | SW | MPW | C | MPC | SC |
| Organic C (%) | 0.233^e^ | 0.015^c^ | 0.300^a^ | 0.007^d^ | 0.024^b^ | 0.006^c^ | 0.038^b^ | 0.023^b^ | 0.047^b^ | 1.207^a^ |
| Available P(mg/L) | 7.404^a^ | 2.953^c^ | 1.902^d^ | 3.170^b^ | 1.105^e^ | 4.787^a^ | 1.426^b^ | 1.771^b^ | 2.377^b^ | 6.009^a^ |
| % Nitrogen | 0.003^b^ | 0.002^b^ | 0.018^a^ | 0.002^b^ | 0.002^b^ | 0.004^b^ | 0.005^b^ | 0.006^a^ | 0.063^a^ | 0.017^b^ |
| K(Cmol/kg) | 0.113^e^ | 1.182^a^ | 0.226^c^ | 0.275^b^ | 0.134^d^ | 2.040^b^ | 5.400^c^ | 27.260^a^ | 2.040^b^ | 2.040^b^ |
| Na (Cmol/kg) | 0.355^c^ | 0.209^e^ | 0.281^d^ | 1.264^a^ | 0.430^b^ | 82.370^d^ | 96.903^c^ | 125.220^a^ | 104.630^b^ | 85.890^d^ |
| Fe(ppm) | 505.000^a^ | 132.500^b^ | 82.500^e^ | 120.000^c^ | 97.500^d^ | 468.860^d^ | 605.830^b^ | 615.570^b^ | 551.060^c^ | 654.440^a^ |
| Cu (ppm) | 0.226^a^ | 0.049^c^ | 0.049^c^ | 0.049^c^ | 0.108^b^ | 22.750^b^ | 30.540^a^ | 14.940^c^ | 22.750^b^ | 17.550^c^ |
| Zn(ppm) | 0.721^a^ | 0.145^b^ | 0.088^c^ | 0.074^d^ | 0.074^c^ | 17.080^d^ | 18.360^d^ | 154.770^a^ | 127.000^b^ | 26.030^c^ |
| Mn (ppm) | 12.805^b^ | 4.672^e^ | 5.058^d^ | 9.698^c^ | 15.144^a^ | 7.920^c^ | 18.540^b^ | 83.760^a^ | 18.553^b^ | 13.220^b^ |
| Mg (Cmol/kg) | 0.129^a^ | 0.045^b^ | 0.033^c^ | 0.027^d^ | 0.015^e^ | 6.990^b^ | 6.990^b^ | 28.030^a^ | 6.990^b^ | 4.590^c^ |
| Ca (Cmol/kg) | 0.128^b^ | 0.085^c^ | 0.173^a^ | 0.042^d^ | 0.085^c^ | 66.970^b^ | 74.280^a^ | 75.740^a^ | 37.543^c^ | 26.160^d^ |
| SO_4_(ppm) | 4.677^d^ | 5.411^c^ | 2.456^e^ | 6.888^b^ | 8.569^a^ | 5.866^c^ | 23.730^a^ | 15.733^b^ | 4.533^c^ | 22.660^a^ |
| NO_3_(ppm) | 13.088^a^ | 5.290^c^ | 6.590^b^ | 5.940^d^ | 13.080^a^ | 0.272^b^ | 1.611^a^ | 0.332^b^ | 2.293^a^ | 2.293^a^ |

**Key: SW – Source of warm spring, MPW – Midpoint of warm spring, C – Confluence of the warm and cold spring, SC – source of cold spring, MPC – Midpoint of cold spring. Organic C. - Organic Carbon, Available P- Available Phosphorus, K – Potassium, Na – Sodium, Fe – Iron, Cu – Copper, Zn – Zinc, Mn –Manganese, Mg – Magnesium, Ca – Calcium, SO_4­_ – Sulphates, NO_3_ – Nitrates. Mean values of duplicate samples with similar letter(s) across rows are significantly different at *P < 0.05* by Duncan Multiple Range Test (DMRT)**

**Table S1b. Mean physicochemical values from water samples obtained at respective sampling points from Ikogosi springs in June and December, 2018**

| **WET SEASON (JUNE) DRY SEASON (DECEMBER)** | | | | | | | | | | | |
| --- | --- | --- | --- | --- | --- | --- | --- | --- | --- | --- | --- |
|  | **SAMPLE SITES** | | | | | **SAMPLE SITES** | | | | | |
| Parameter | SW | MPW | C | MPC | SC | SW | MPW | C | MPC | SC |  |
| Temp.(°C) | 38.000^a^ | 37.330^a^ | 28.330^b^ | 29.000^b^ | 28.330^b^ | 38.330^a^ | 37.330^a^ | 29.670^b^ | 25.670^c^ | 26.670^c^ |  |
| pH | 6.360^d^ | 7.930^a^ | 7.430^b^ | 7.170^c^ | 7.070^c^ | 5.800^b^ | 7.400^a^ | 7.600^a^ | 7.400^a^ | 7.600^a^ |  |
| DO (ppm) | 7.060^b^ | 7.000^c^ | 7.220^a^ | 6.540^d^ | 6.250^e^ | 6.840^b^ | 8.020^a^ | 6.450^b^ | 7.250^ab^ | 7.150^ab^ |  |
| TDS (ppm) | 42.013^a^ | 36.000^b^ | 22.000^c^ | 20.967^c^ | 18.000^d^ | 40.330 | 42.330 | 32.330 | 42.330 | 36.330 |  |
| K(ppm) | 0.449^a^ | 0.427^b^ | 0.326^c^ | 0.312^d^ | 0.283^e^ | 0.840^a^ | 0.840^a^ | 0.840^a^ | 0.580^b^ | 0.580^b^ |  |
| Na (Cmol/kg) | 28.022^b^ | 30.111^a^ | 20.011^d^ | 21.962^c^ | 9.027^e^ | 0.040 | 0.070 | 0.040 | 0.070 | 0.070 |  |
| Fe(ppm) | 1.750^a^ | 1.750^a^ | 1.500^b^ | 1.001^c^ | 1.000^c^ | 0.320^c^ | 0.560^ab^ | 0.320^c^ | 0.480^b^ | 0.640^a^ |  |
| Cu (ppm) | 0.005 | 0.005 | 0.005 | 0.005 | 0.344 | 0.060 | 0.060 | 0.040 | 0.040 | 0.040 |  |
| Zn(ppm) | 0.003^b^ | 0.005^a^ | 0.003^b^ | 0.003^b^ | 0.003^b^ | 0.090^a^ | 0.040^c^ | 0.040^c^ | 0.080^ab^ | 0.050^bc^ |  |
| Mn (ppm) | 0.121^a^ | 0.830^b^ | 0.044^c^ | 0.044^c^ | 0.086^b^ | 0.060^b^ | 0.150^ab^ | 0.060^b^ | 0.160^a^ | 0.063^b^ |  |
| Mg (Cmol/kg) | 1.023^b^ | 1.133^a^ | 0.586^c^ | 0.476^d^ | 0.295^e^ | 3.410^b^ | 8.040^a^ | 3.020^b^ | 3.140^b^ | 3.140^b^ |  |
| Ca (Cmol/kg) | 18.152^a^ | 15.807^b^ | 10.304^c^ | 6.272^d^ | 6.272^d^ | 11.000^b^ | 10.210^b^ | 9.010^b^ | 11.000^b^ | 16.170^a^ |  |
| SO_4_(ppm) | 3.806^a^ | 3.174^b^ | 2.574^e^ | 2.658^c^ | 2.674^c^ | 2.130^ab^ | 1.680^bc^ | 1.070^c^ | 1.240^c^ | 2.670^a^ |  |
| NO_3_(ppm) | 4.040^c^ | 3.540^a^ | 3.350^d^ | 3.350^d^ | 5.940^b^ | 2.750^d^ | 2.550^d^ | 3.140^c^ | 3.850^b^ | 4.440^a^ |  |
| Electrical  Conductivity(S/m) | 85.037^a^ | 72.000^b^ | 45.133^c^ | 44.000^c^ | 38.000^d^ | 82.330^a^ | 84.670^a^ | 64.330^b^ | 83.670^a^ | 74.000^c^ |  |

**WHO LIMITS: Temperature (22.00-32.00), pH (6.50-8.50), DO (<5.00), TDS (500), K (30.00), Na (200.00), Fe (0.50 - 50.00), Cu (2.00), Mg (200.00), Ca (200.00), SO_4_ (100.00), NO_3_ (<50.00), Electrical conductivity (<1000.00).**

**Key: SW – Source of warm spring, MPW – Midpoint of warm spring, C – Confluence of the warm and cold spring, SC – source of cold spring, MPC – Midpoint of cold spring. Temp. – Temperature, DO – Dissolved Oxygen, TDS – Total Dissolved Solids, K – Potassium, Na – Sodium, Fe – Iron, Cu – Copper, Zn – Zinc, Mn –Manganese, Mg – Magnesium, Ca – Calcium, SO_4­_ – Sulphates, NO_3_ – Nitrates. Mean values of duplicate samples with similar letter(s) across rows are not significantly different at *P < 0.05* by Duncan Multiple Range Test (DMRT)**

**Table S2: Summary of fungal taxonomic classification identified using UNITE database**

| **Sample groupings** |  | **FUNGAL TAXONOMIC CLASSIFICATION** | | | | | |
| --- | --- | --- | --- | --- | --- | --- | --- |
|  |  | **Phylum** | **Class** | **Order** | **Family** | **Genus** | **Species** |
| **Sample Types** | **Water** | **5** | **24** | **66** | **161** | **306** | **370** |
|  | **Sediment** | **4** | **14** | **33** | **58** | **275** | **91** |
|  |  |  |  |  |  |  |  |
| **Month of Collection** | **June** | **4** | **19** | **52** | **117** | **177** | **191** |
|  | **December** | **6** | **25** | **70** | **171** | **327** | **400** |
| **Sample types and** | **June Sediment** | **4** | **19** | **52** | **117** | **177** | **191** |
| **Month of collection** | **June Water** | **3** | **16** | **41** | **103** | **152** | **167** |
|  | **December Sediment** | **6** | **20** | **52** | **127** | **217** | **248** |
|  | **December Water** | **5** | **23** | **64** | **149** | **275** | **317** |
| **Spring Location** | **Source of warm spring (SW)** | **4** | **14** | **33** | **58** | **78** | **91** |
|  | **Midpoint of warm spring (MPW)** | **3** | **14** | **26** | **58** | **73** | **81** |
|  | **Confluence (C)** | **5** | **18** | **50** | **125** | **219** | **237** |
|  | **Midpoint of cold spring**  **(MPC)** | **6** | **23** | **62** | **148** | **270** | **307** |
|  | **Source of cold spring (SC)** | **6** | **22** | **63** | **146** | **268** | **304** |

**TABLE S3: Breakdown of ITS1 reads before and after quality preprocessing using DADA2 pipeline**

| **Sample name** | **Raw reads** | **Filtered reads** | **Denoised reads** | **Non-chimeric reads** |
| --- | --- | --- | --- | --- |
| SW-water-dry | 38955 | 38813 | 38811 | 38735 |
| SW-water-wet | 47109 | 47089 | 47085 | 47085 |
| SW-sediment-dry | 38153 | 37952 | 37938 | 37938 |
| SW-sediment-wet | 38430 | 38163 | 38089 | 38018 |
| MPW-water-dry | 38510 | 38369 | 38290 | 38290 |
| MPW-water-wet | 39546 | 39229 | 39228 | 39228 |
| MPW-sediment-dry | 37856 | 37607 | 37605 | 37605 |
| MPW-sediment-wet | 38321 | 38020 | 37995 | 37882 |
| C-water-dry | 39484 | 39365 | 39318 | 39318 |
| C-water-wet | 36565 | 36106 | 35952 | 35952 |
| C-sediment-dry | 41335 | 41114 | 41054 | 40939 |
| C-sediment-wet | 38390 | 38079 | 38072 | 38032 |
| MPC-water-dry | 39317 | 39127 | 38974 | 38974 |
| MPC-water-wet | 39780 | 39557 | 39490 | 39365 |
| MPC-sediment-dry | 39273 | 39002 | 38988 | 38803 |
| MPC-sediment-wet | 41145 | 40949 | 40920 | 40920 |
| SC-water-dry | 39071 | 38840 | 38445 | 38370 |
| SC-water-wet | 38986 | 38717 | 38713 | 38713 |
| SC-sediment-dry | 41127 | 40892 | 40870 | 40870 |
| SC-sediment-wet | 45319 | 45066 | 45054 | 45054 |
|  | **796,672** | **792,056** | **790,891** | **790,091** |

**Key: SW – Source of warm spring, MPW – Midpoint of warm spring, C – Confluence of the warm and cold spring, SC – source of cold spring, MPC – Midpoint of cold spring.**

Table S1 shows a total of 796,672 high-quality reads which were preprocessed using DADA2 (version 1.18). These reads were subjected to the initial filtering stage, yielding 792,056 filtered reads. Further quality control step – denoising, resulted in a slightly lower number of reads (790,891). Lastly, these denoised sequences were filtered to remove chimeric reads to produce only high-quality non-chimeric reads (790,091). These cleaned non-chimeric reads were thereafter used for downstream processing, including taxonomic classification, alpha diversity, and redundancy analysis. The total percentage of reads that survived the quality processing stage was 99.17%.

**S2 Phyla Composition of Ikogosi warm springs**

Samples (water and sediment) collected in June had relative phyla abundance of 63.45% and 36.41% distributed amongst the Ascomycota and Basidiomycota, respectively. The dominance of the Ascomycota was correspondingly observed in December samples, with Ascomycota constituting 79.28% and Basidiomycota 20.01% (Figure S1).

Figure S1. Taxonomic compositional bar plot of fungal phyla identified in December and June (from water and sediment) samples of Ikogosi warm springs.

At the phylum-level, Ascomycota was the most dominant phyla in both water and sediment samples comprising 74.64% and 66.53% respectively, followed by Basidiomycota with 24.78% and 34.27% abundance respectively (Figure S2).

Figure S2A. Taxonomic compositional bar plot of fungal phyla from sediment and water samples of Ikogosi warm springs

In Figure S2B, water samples depicted high relative abundance of *Meyerozyma* (27.47%)*, Trichosporon* (15.22%) and *Penicillium* (5.31%) than its corresponding sediment samples which depicted *Meyerozyma* (1.09%)*, Trichosporon* (1.87%) and *Penicillium* (1.87%). However, some genera such as Malassezia (12.84%) *Condenascus* (11.54%), *Apiotricihum* (5.90%) and *Cladosporium* (5.34%), were discovered in sediment samples in high prevalence than in water samples (Malassezia – 1.17%, *Cladosporium –* 1.19%, *Apiotrichum -* 0.08% and *Condenascus* – 0%). Interestingly, *Aspergillus* was predominant in both samples comprising 11.69% and 14.04% in sediment and water respectively.

~~
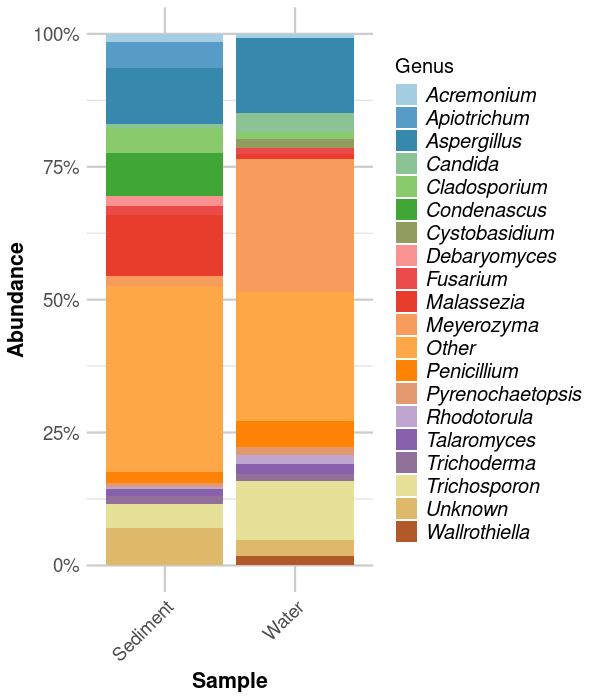
~~

Figure S2B.Taxonomic compositional bar plots showing the most abundant fungal genera from sediment and water samples of Ikogosi warm springs.

Comparing the seasonal fungal diversity of all samples, water samples collected in December (WD) showed highest prevalence of Ascomycota (89.02%), albeit they were prevalent in all samples across all seasons, followed by Basidiomycota whose lowest abundance was in WD (10.68 %) (Figure S3).

Figure S3. Taxonomic compositional bar plot of fungal phyla detected from (sediment and water) samples collected in December and June from Ikogosi warm springs

With regard to diversity from different locations of the spring, the midpoint of the warm spring was mostly dominated by phylum Ascomycota (83.71%), whilst the least relative abundance of Ascomycota (55.43%) was recorded at the source of the warm spring (SW) (Figure S4). However, the source of the warm spring had the largest relative abundance of Basidiomycota (44.51%) while the midpoint of the warm spring (MPW) depicted the least abundance of Basidiomycota recorded as 16.28%. Neither Basidiobolomycota nor Monoblepharomycota were discovered from all sampled sites.

Figure S4. Taxonomic compositional bar plot of fungal phyla detected at different sampling points of Ikogosi warm springs

**S3 Alpha Diversity**

| ~~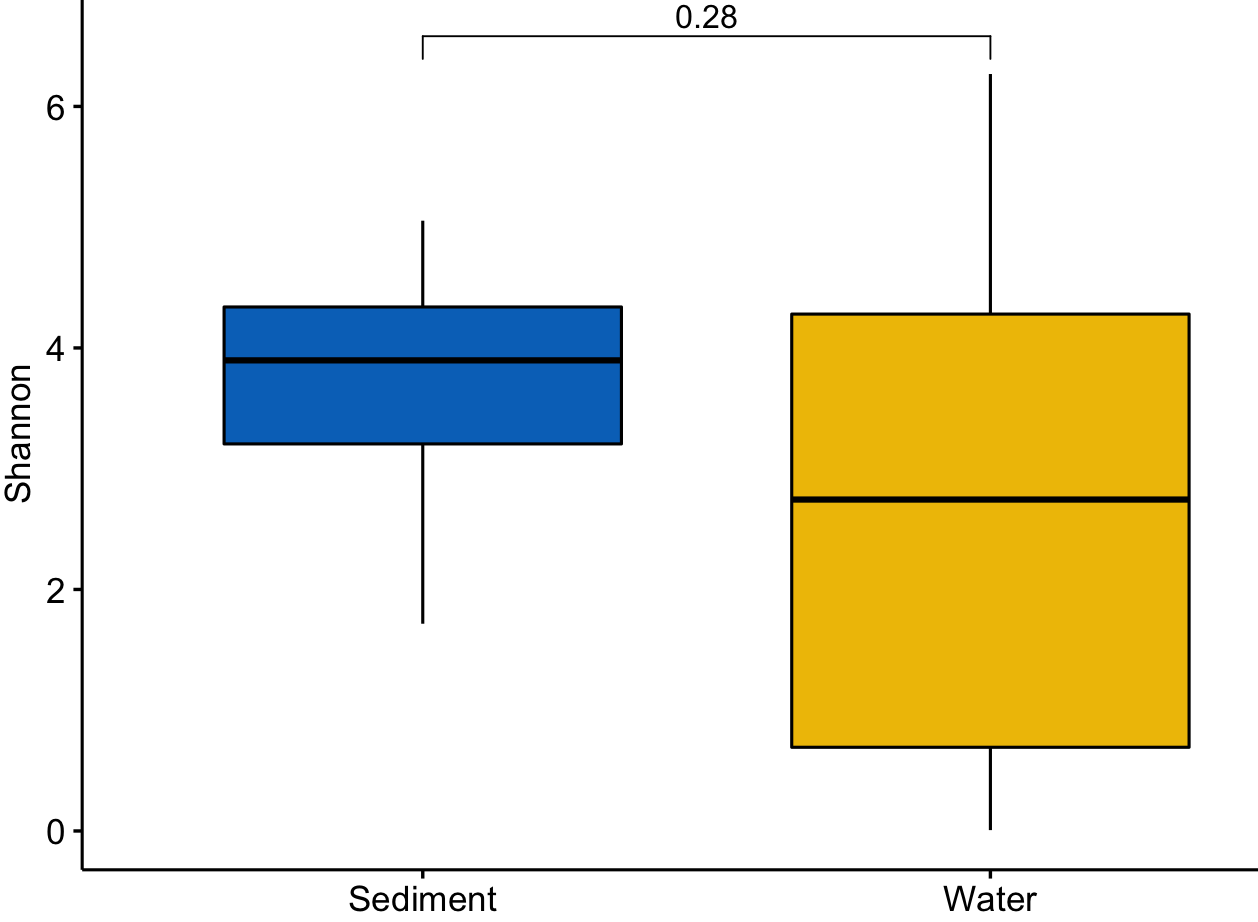~~ | ~~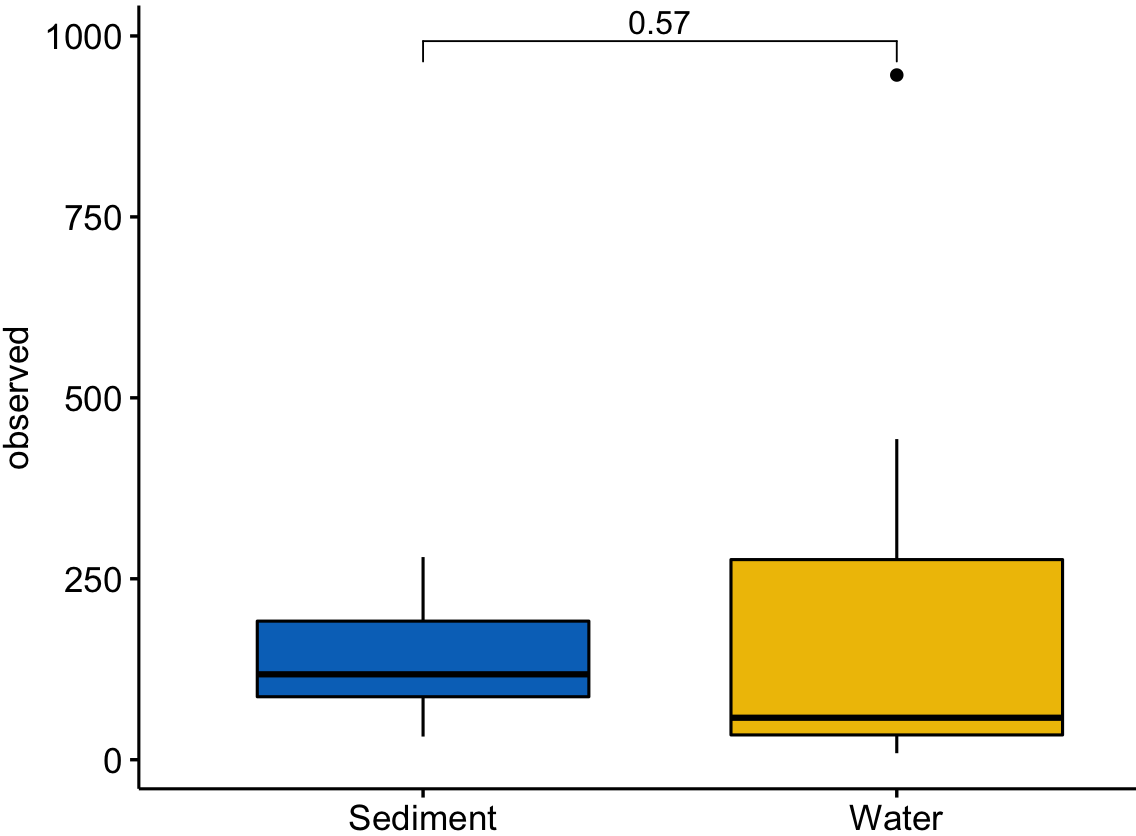~~ |
| --- | --- |

Figure S5: Fungal alpha diversity boxplots of samples collected from the sediment and water (from all sampled sites) of Ikogosi warm springs using (a) Shannon and (b) Observed species index (P<0.05). Shannon and observed metrics indicating less species diversity and community richness in sediment samples than in water samples. At *P* < 0.05, this observation was statistically insignificant recording p values of 0.28 and 0.57 respectively.

**S4 Redundancy Analysis**

Redundancy analysis was performed to examine the relationship between the fungal communities in sediment samples and their environmental parameters. In RDA for sediments,

RDA1 and RDA2 explained 12.9% and 11.6% of the variations observed in the fungal community of sediment samples respectively (Figure S6). These two axes describe only approximately 25% of the overall variation, signifying that other contributing factors may also have played a role in the observed pattern. RDA postulates that fungal association in sample SC1_sediment_dry is strongly associated with organic carbon. This sample was collected from the source of the cold spring during the dry season. This parameter did not seem to affect its counterpart wet sample (SC1_sediment_wet). In particular, sulphates seemed to have a great effect on the fungal community present in the sample labelled MPC1_sediment_dry; sediment sample collected from the midpoint of the cold spring during the dry season. Lastly, higher calcium seemed to have an effect on the fungal communities present in SW1-sediment-wet and SW1-sediment-dry. These samples originated from the source of the warm spring collected during the wet and dry seasons, respectively.

s
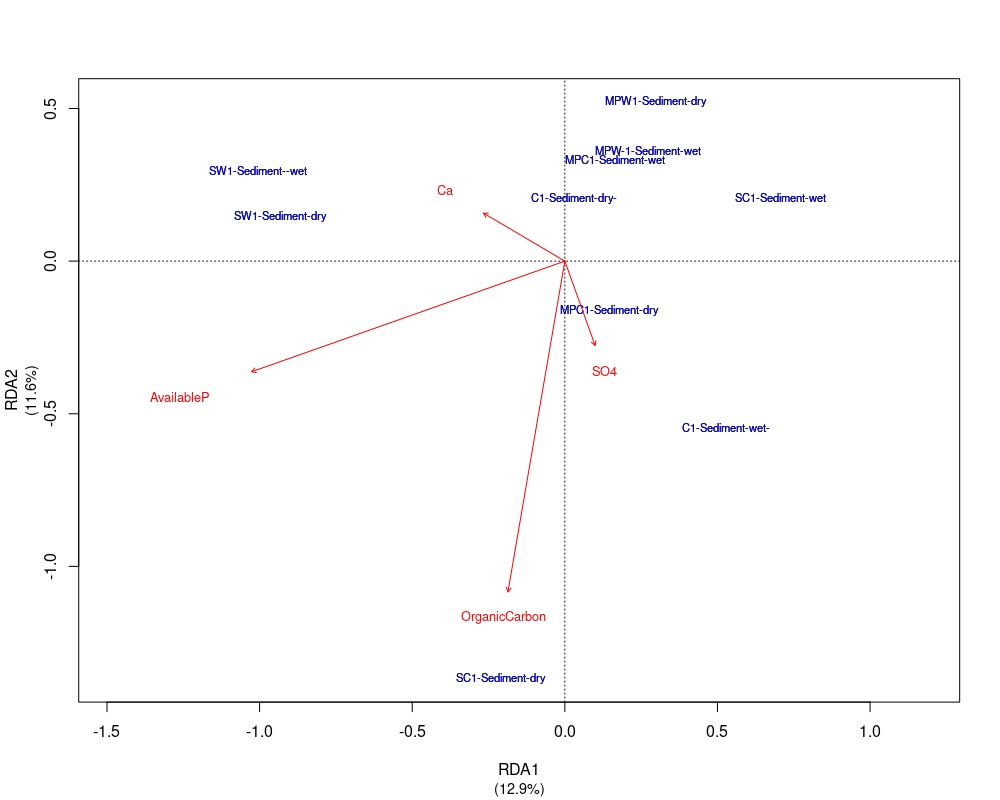


Figure S6. Redundancy analysis (RDA) demonstrating sediment fungal communities constrained by some sediment physicochemical properties. Ca (Calcium), available phosphorus, SO_4_ (sulphates), and organic carbon. The first two RDA axes explain 24.7% of the fungal community and environmental association. Each label represents a sample, named by its site of collection and season. Red arrows represent each tested environmental parameter (p < 0.05, one-way anova permutation test perormed 999 times).

For water samples, redundancy analysis was performed using only environmental parameters that were significant at p <0.05 using one-way anova permutation tests, performed 999 times Figure S7). These environmental parameters were temperature, total dissolved solids, potassium, sulphates, and pH. RDA1 and RDA2 accounted for 23.1% and 16.8% of the variations observed in the fungal association-environmental relationship, respectively, resulting in approximately 40% of the total observed pattern. RDA demonstrated that samples showed a distinct seasonal separation, with wet season samples (MPW1-Water-wet, SC-Water-wet, C1-Water-wet, MPC-Water-wet, and SW1-Water-wet) clustered on the positive side of the plot along RDA 1. Arrow lengths showed sulphate (SO₄) as the strongest positive correlation with wet-season samples. The majority of dry season samples (MPW1-water-dry, SW1-water-dry, and C1-water-dry) were scattered across the left side of the plot, with TDS, temperature, and potassium showing correlations with only dry season samples.

s
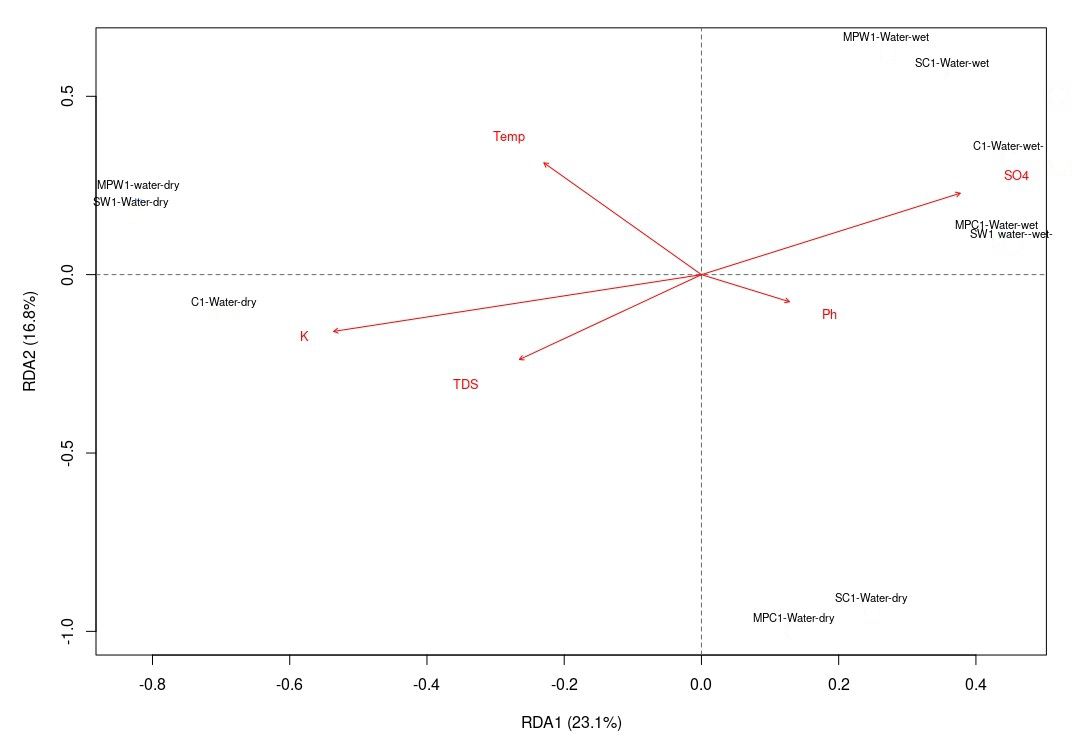


Figure S7. Redundancy analysis (RDA) demonstrating fungal communities from water samples constrained by some water physicochemical properties. TDS (Total dissolved solids), Potassium, temperature, SO_4_ (sulphates), and pH. The first two RDA axes explained 39.9% of the fungal community and environmental association. Each label represents a sample, named by its site of collection and season. Red arrows represent each tested environmental parameter (p < 0.05, one-way anova permutation test performed 999 times).

 In addition, Fusarium can be found in aquatic habitats, including sea-

water, river water (Palmero etal.

In addition, Fusarium can be found in aquatic habitats, including sea-

water, river water (Palmero etaThe relative abundance of Proteobacteria, Cyanobacteria showed a positive

correlation with moderate temperature

The relative abundance of Proteobacteria, Cyanobacteria shows
